# Chemoselective Reduction of Fenofibric Acid to Alcohol in the Presence of Ketone by Mixed Anhydride and Sodium Borohydride

**DOI:** 10.4236/ijoc.2022.122010

**Published:** 2022-06-30

**Authors:** Greesha N. Majethia, Wahajul Haq, Ganesaratnam K. Balendiran

**Affiliations:** Department of Chemistry, Youngstown State University, Youngstown, OH, USA.

**Keywords:** Chemoselective, Reduction of Carboxylic Acid, Sodium Borohydride, Mixed Anhydride

## Abstract

A highly efficient and facile protocol for the selective reduction of carboxylic acid of Fenofibric acid to corresponding alcohol was developed. The selective reduction was carried out by activation of carboxylic acid by mixed anhydride followed by the reaction of sodium borohydride in presence of methanol. This is the first example of chemoselective reduction of carboxylic acid to alcohol in presence of a ketone without any external catalyst or ligand in a single step. The reaction offers wide applicability for the selective carboxylic group reduction methodology. The chemoselective reduction was demonstrated by the reduction of Fenofibric acid, an active metabolite of the drug Fenofibrate, to corresponding alcohol in excellent selectivity, yield, and purity.

## 1. Introduction

The chemistry of the carbonyl group has been extensively used in various synthetic transformations. The carbonyl chemistry comprises a repertoire of several name reactions and reagents used for a specific purpose. The reduction of carbonyl compounds to other functional groups offers new synthetic applications and the identification of novel chemical molecules. The conversion of a carbonyl group to corresponding alcohol is one of the important steps studied by various researchers. Several reagents have been developed for this purpose, such as LiAlH4, NaBH4, and DIBAL-H, Red-Al, and other combinations [1]–[7]. There are reports of selective reduction of aldehydes in presence of other carbonyl compounds including ketones leading to atom economy and overall yield improvement [8] [9] [10] [11].

The reduction of carboxylic group to alcohols is tricky and requires strong conditions for the reduction as compared to aldehydes, ketones and ester groups. The functional group tolerance is also compromised and therefore there is a need for highly efficient reducing agent under mild reaction conditions. This has been achieved by the transformation of carboxylic group to a highly reactive intermediate which in turn is reduced by a mild reducing agent like sodium borohydride. It has been reported that the activated carboxylic acid can be successfully reduced to corresponding alcohols by the reaction of sodium borohydride at room temperature in excellent yield. The reduction of an activated carboxylic acid is reported using sodium borohydride by carbonates or mixed anhydrides, O-acylurea generated by *in situ* action of carbodiimides, and activated esters and boronic esters [12]–[22].

Selective and orthogonal reduction of carbonyl compounds remains an important area of active research. Fairly good chemoselectivity has been achieved with the advancement of methodology however, there is still a need for robust methodology to achieve the selectivity for many combinations for industrial application. Among various combinations, the orthogonal reduction of carboxylic acids is reported in the presence of derivatives like esters and amides, to mention a few. Catalytic hydrosilylation has been shown to be a very useful approach for the reduction of carboxylic acid to alcohol. The available methodology and future prospects were reviewed in consideration of the significance of orthogonal selectivity and chemoselectivity by catalytic hydrosilylation of carboxylic acid derivatives under mild conditions [23].

Recently, a chemoselective reduction was reported using hydrosilylation and combination with [Ni-OH] complex as a catalyst for the reduction of amide to amines under base-free conditions with key functional group tolerance [24]. The hydroboration reduction of carboxylic acids using sodium amino diborane as a catalyst has been reported in excellent selectivity of functional group tolerance [25]. Therefore, a facile reduction of carboxylic acid with functional group tolerance is highly desirable.

**Figure 1.**
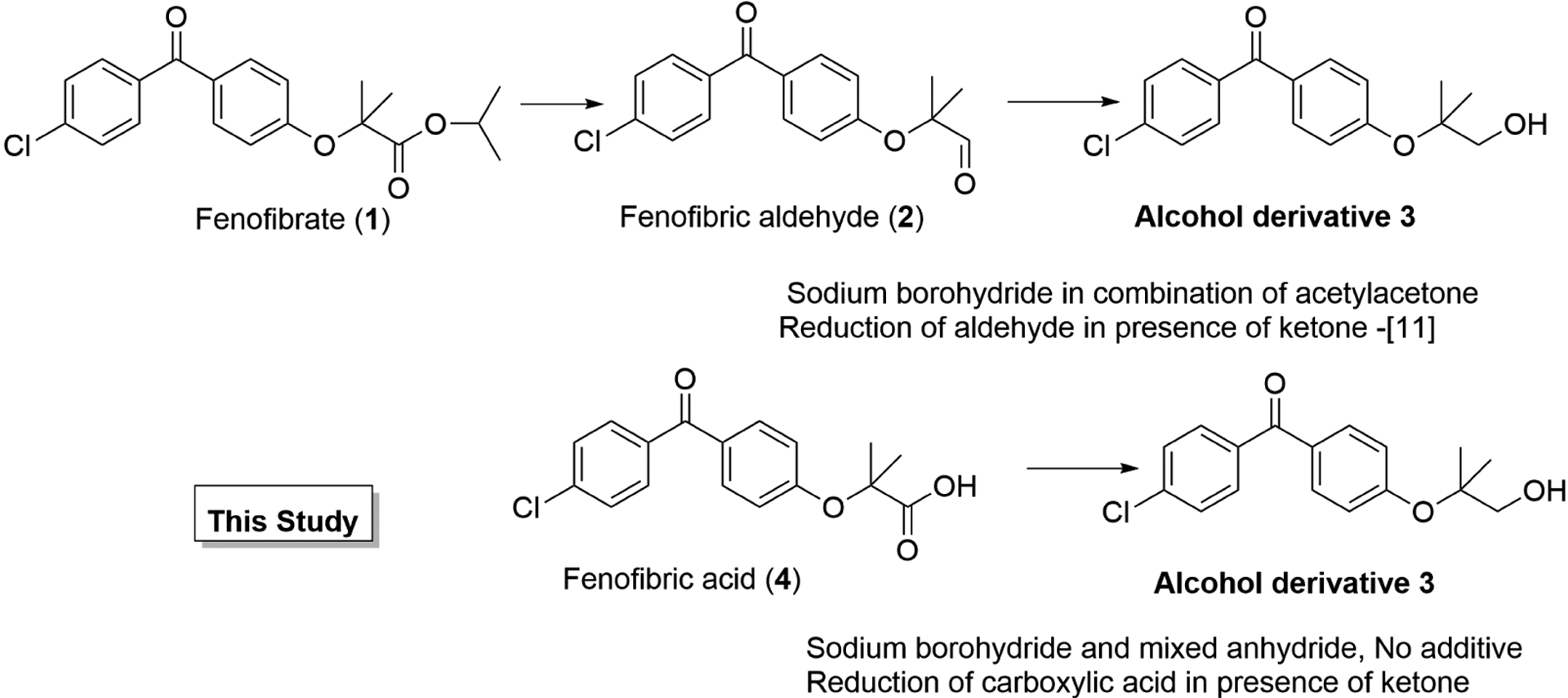
Direct reduction of carboxylic acid in presence of ketone.

Herein we report a facile and chemoselective reduction of a carboxylic group, more challenging carbonyl group, to corresponding alcohol in presence of a ketone under mild condition without any external ligand or catalyst in excellent yield and selectivity. In our ongoing research project on Fibrates we envisaged the synthesis of alcohol derivatives(4-chlorophenyl)(4-((1-hydroxy-2-methylpropan-2-yl) oxy)phenyl)methanone (**3**) [26] [27] [28]. The synthesis of the title compound can be achieved by the two step reaction as reported recently that involves double hydride reduction of the Fenofibrate, an agent against diabetes and that lowers lipid levels [29] [30] [31] [32] [33]. The isopropyl group of the Fenofibrate was reduced to corresponding aldehyde **2** by the reduction with DIBAL-H and subsequently the chemoselective reduction of the aldehyde group was achieved to obtain **3** using sodium borohydride in presence of ketone group using acetylacetone as ligand masking the ketone group of benzophenone moiety [11] Figure 1.

In order to develop a facile and direct synthesis of **3** under mild conditions, we surmised that reduction of an activated carboxylic group by commonly accessible and bench stable sodium borohydride may be useful for a chemoselective reduction in the presence of another carbonyl group, a ketone, in the current study. The chemoselectivity may be attributed to the relative reactivity of an activated carboxylic acid and other co-existing carbonyl groups. For the present study, we have selected mixed anhydride using isobutyl chloroformate in presence of a tertiary amine at optimum temperature for mixed anhydride reaction ranging from −15°C to −10°C. The selective reactivity of sodium borohydride on the activated carboxylic group and other co-existing carbonyl groups may be more evident at the low-temperature range used for mixed anhydride reactions. The mixed anhydride is generated by the reaction of Fenofibric acid (**4**) with iso-butyl chloroformate and N-methyl morpholine in dry Tetrahydrofuran at −15°C to −10°C. The mixed anhydride thus obtained was treated with 1.5 to 2 molar equitant of sodium borohydride at −15°C followed by the addition of methanol. The reaction was quenched after 5 min by the addition of 1 N hydrochloric acid below −10°C and the product was isolated by a usual workup.

The reaction product came out as per the expectation as evident from the thin layer of chromatography on the silica gel plate. The reaction product showed a chromatographically homogeneous single spot and the starting material was completely consumed. The reaction product was characterized by the distinctive peak corresponding to alcoholic methylene singlet in NMR as desired for compound **3** and the absence of carboxylic carbonyl in the ^13^C NMR (see experimental).

### Reagents and Conditions

- N-methyl morpholine and isobutyl chloroformate −15°C, 5 min.
- Stoichiometric NaBH_4_ in methanol −15°C, 5 min.

**Figure 2.**
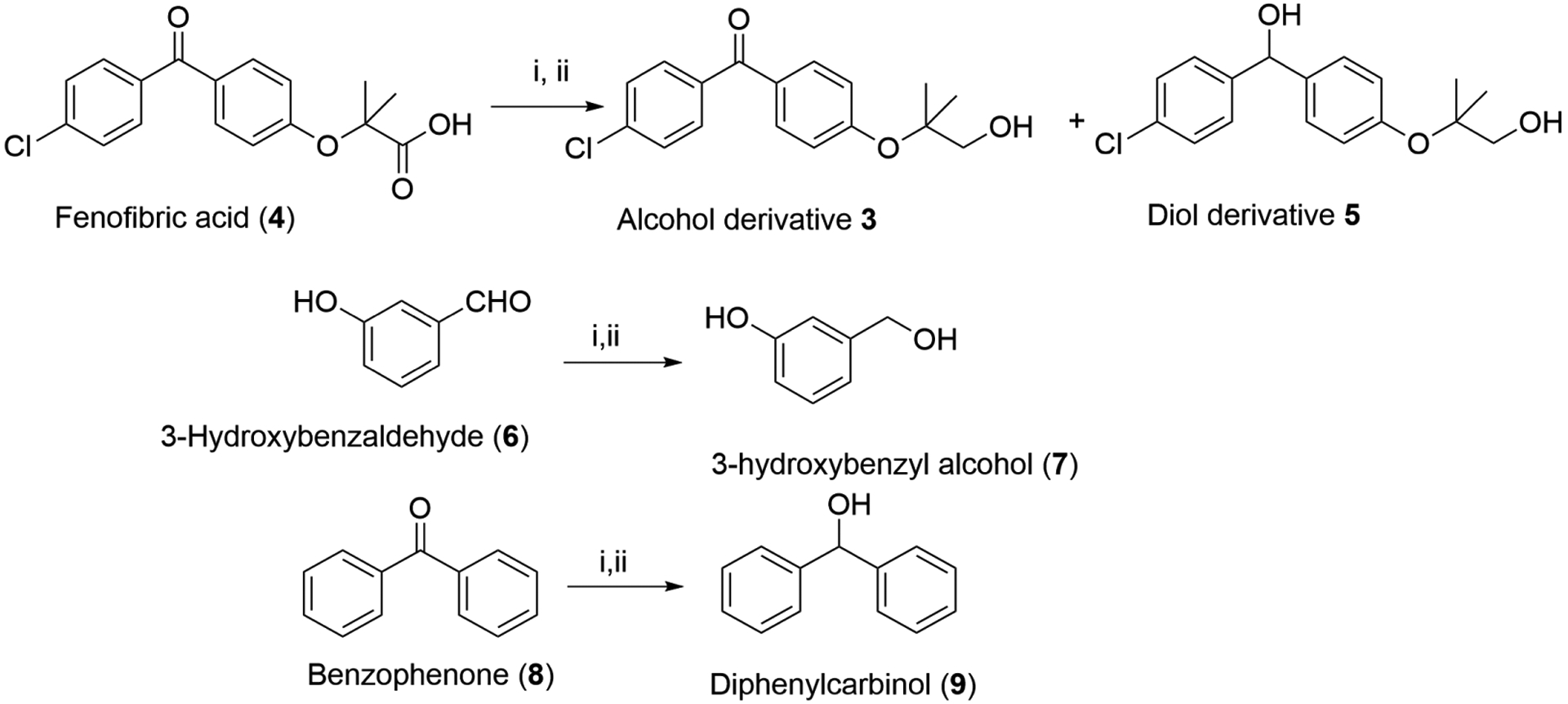
Reactions for optimization studies.

The details of experimental conditions of the reaction optimization studies are summarized in Figure 2 and Table 1. It is apparent from the results that activation of the carboxylic group by the mixed anhydride followed by the reaction of sodium borohydride resulted in the formation of the desired alcohol 3 as the exclusive product. The stoichiometric excess of sodium borohydride has little impact as the use of excess equivalents has an insignificant effect on the selectivity of the reaction and the best combination was the use of a 2 molar excess of sodium borohydride (Entry-2).

The reaction time did not impact on selectivity of the reaction as no change was observed when the reaction was allowed to continue extra time with maintaining the temperature below −10°C as shown in entries 5 and 6 in the optimization table. The reduced product corresponding to diol was not visible on the TLC plate but can be seen in NMR as one additional singlet at 5.89 ppm corresponding to reduced ketone. The quantities given in table for the % yield are based on the proton NMR of the crude products without purification and the values are drawn by the signal intensity of the signature peak of hydroxyl methylene protons as singlet. It is important to note that the reaction temperature seems to play a significant role in maintaining selectivity. The formation of diol was only observed when the reaction was quenched at room temperature. It is clear from the data that the use of a 2 molar excess of sodium borohydride at −15°C for 5 min is the most appropriate experimental condition for the selective reduction of carboxylic acid to the alcohol. Subsequently, we performed the sodium borohydride reduction of aldehyde and ketone containing compounds in order to validate the role of temperature on the reducing ability of sodium borohydride as shown in Figure 2. The reaction was performed at the optimized reaction condition viz. use of 2 molar excess of sodium borohydride at −15°C for 5 mins. During these reactions, we have observed mixed results. The aldehyde **6** was completely reduced to corresponding alcohol **7** whereas the reduction of ketone **8** was incomplete and the conversion was around 50% only (Entries 9 & 10). The data of Table 1 that temperature plays a significant role in selectivity but the reactivity of the mixed carbonic anhydride seems to play a decisive role in chemoselectivity.

Synthesis of (4-chlorophenyl)(4-((1-hydroxy-2-methylpropan-2-yl)oxy) phenyl) methanone (**3**) has recently been reported in a recent communication by chemoselective reduction of aldehyde in presence of a ketone [11]. Chemoselective reduction of aldehydes was reported with a combination of acetylacetone and NaBH_4_ under mild conditions by a double selective reduction procedure. However, in the present study, we have developed a highly efficient and direct route to alcohol **3** by the chemoselective reduction of carboxylic acid, more difficult to reduce, under mild conditions. The Chemoselective reduction of carboxylic acid in presence of a ketone has potential of wide application in the area of medicinal chemistry for the synthesis of complex molecules.

## 2. Experimental

The synthesis of **3** was carried out starting from the Fenofibrate obtained from Sigma USA. The Fenofibrate was converted the Fenofibric acid (**4**) by alkaline hydrolysis according to the method reported in the literature. Fenofibric acid (4) 318 mg (1 mmole) was taken in dry THF (8 ml) in an oven-dried, 50 mL round-bottomed flask with a magnetic stir-bar and moisture guard tube. The solution was kept under stirring at −15°C in acetone: dry ice bath. To this chilled and stirred solution N-methyl morpholine, 0.11 ml (1.1 mmole) was added followed by the addition of isobutyl chloroformate 0.14 ml (1.1 mmole). The stirring was continued for 4 – 5 min maintaining the reaction temperature at −15°C. After 5 minutes sodium borohydride 80 mg (2 mmole) was added to the reaction mixture followed by the dropwise addition of methanol (1 ml). The reaction was stirred maintaining the reaction temperature strictly at −15°C for 5 minutes followed by the quenching of the reaction by the addition of 2 ml of 1N hydrochloric acid to the reaction mixture. The reaction mixture was allowed to reach room temperature and the solvent was removed under reduced pressure. The residue was taken in ethyl acetate 20 ml and washed with brine until neutral to pH. The organic layer was dried over anhydrous magnesium sulfate and filtered using a Whatman #1 filter paper. The solvent was removed under reduced pressure to obtain the chromatographically homogeneous product as white solid and the spectral data was in agreement with the literature [11].

## 3. Synthesis of (4-Chlorophenyl)(4-((1-Hydroxy-2-Methylpropan-2-Yl) Oxy)Phenyl)Methanone (3)

The synthesis of 3 was carried out starting from the Fenofibrate obtained from Sigma USA. The Fenofibrate was converted to the Fenofibric acid by alkaline hydrolysis according to the method reported in the literature [34]. Fenofibric acid (**4**) 318 mg (1 mmole) was taken in dry THF (8 ml) in an oven-dried, 50 mL round-bottomed flask with a magnetic stir-bar and moisture guard tube. The solution was kept under stirring at −15°C in acetone: dry ice bath. To this chilled and stirred solution N-methyl morpholine, 0.11 ml (1.1 mmole) was added followed by the addition of isobutyl chloroformate 0.14 ml (1.1 mmole). The stirring was continued for 4 – 5 min maintaining the reaction temperature at −15°C. After 5 minutes Sodium Borohydride 80 mg (2 mmole) was added to the reaction mixture followed by the dropwise addition of methanol (1 ml). The reaction was stirred maintaining the reaction temperature strictly at −15°C for 5 minutes followed by the quenching of the reaction by the addition of 2 ml of 1N hydrochloric acid to the reaction mixture. The reaction mixture was allowed to reach room temperature and the solvent was removed under reduced pressure. The residue was taken in ethyl acetate 20 ml and washed with brine until neutral to pH. The organic layer was dried over anhydrous magnesium sulfate and filtered using a Whatman #1 filter paper. The solvent was removed under reduced pressure to obtain the chromatographically homogeneous product as white solid. Yield 285 mg, 94%. M.P. 137°C - 138°C, ^1^H NMR (400 MHz, CDCl_3_): *δ* = 7.75 – 7.71 (m, 4H), 7.45 (d, J = 8.6 Hz, 2H), 7.07 (d, J = 8.7 Hz, 2H), 3.63 (s, 2H), 1.37 (s, 6H). ^13^C NMR: (100 MHz, CDCl_3_) *δ* = 23.0, 59.20, 62.06, 70.58, 77.02, 77.33, 81.88, 122.32, 122.65, 128.59, 131.60, 131.25, 131.72, 131.99, 136.15, 138.57, 159.31, 194.48. IR spectrum (cm^−1^): 2976, 1757, 1245, 1146, 901. Mass Calculated for C_17_H_17_ClO_3_ [M + H]^+^ = 305.7 Observed 305, [M + Na]^+^ = 327.

For reaction optimization studies the reactions were carried out in the same manner as described above but the molar excess of sodium borohydride was taken and reaction time and reaction temperature was adjusted according to Table 1. The crude products were analyzed by TLC and NMR for the quantitation of signature peaks for the formation of compound **3** and compound **5** respectively.

## 4. Synthesis of 3-Hydroxy Benzyl Alcohol (7)

3-hydroxy benzaldehyde (**6**) 112 mg (1 mmole) was taken in dry THF (5 ml) in an oven-dried, 50 mL round-bottomed flask with magnetic stir-bar and moisture guard tube. The solution was kept under stirring at −15°C in acetone: dry ice bath. To this chilled and stirred solution Sodium Borohydride 80 mg (2 mmole) was added to the reaction mixture followed by the dropwise addition of methanol (1 ml). The reaction was stirred maintaining the reaction temperature strictly at −15°C for 5 minutes followed by the quenching of the reaction by the addition of 2 ml of 1N hydrochloric acid to the reaction mixture. The reaction mixture was allowed to reach room temperature and the solvent was removed under reduced pressure. The residue was taken in ethyl acetate 20 ml and washed with brine until neutral to pH. The organic layer dried over anhydrous magnesium sulfate and filtered using a Whatman #1 filter paper. The solvent was removed under reduced pressure to obtain the chromatographically homogeneous product gummy solid. The TLC showed complete conversion of the compound **6** to the reduce product **7** which was obtained as exclusive product. 120 mg, 97%; ^1^H NMR (400 MHz, CDCl_3_): *δ* = 7.25 (t, 2H, J = 7.7 Hz), 6.90 (t, 1H J = 7.7 Hz), 6.76 (m, 1H), 4.65 (s, 2H). ^13^C NMR: (100 MHz, CDCl_3_) *δ* = 155.83, 142.68, 129.87, 119.16, 114.59, 113.74, 77.32, 77.01, 76.69, 65.07. Mass Calculated for C_7_H_8_O_2_ 124.14, Observed [M + H]^+^ = 125.2, 107.

## 5. Synthesis of Diphenyl Carbinol (9)

Benzophenone (**8**) 182 mg (1 mmole) was taken in dry THF (5 ml) in an oven-dried, 50 mL round bottomed flask with magnetic stir-bar and moisture guard tube. The solution was kept under stirring at −15°C in acetone: dry ice bath. To this chilled and stirred solution Sodium Borohydride 80 mg (2 mmole) was added to the reaction mixture followed by the dropwise addition of methanol (1 ml). The reaction was stirred maintaining the reaction temperature strictly at −15°C for 5 minutes followed by the quenching of the reaction by the addition of 2 ml of 1N hydrochloric acid to the reaction mixture. The reaction mixture was allowed to reach room temperature and the solvent was removed under reduced pressure. The residue was taken in ethyl acetate 20 ml and washed with brine until neutral to pH. The organic layer dried over anhydrous magnesium sulfate and filtered using a Whatman #1 filter paper. The solvent was removed under reduced pressure to obtain the chromatographically homogeneous product gummy matter solid. The TLC showed partial conversion of compound **8** to the reduced product **9** as evident from the thin layer chromatography and the signature peaks in the proton NMR. 73 mg, 40%. ^1^H NMR (400 MHz, CDCl_3_): *δ* = 7.55 – 7.20 (m, 10H Aromatic protons), 5.84 (s, 2H, hydroxyl methylene). ^13^C NMR: (100 MHz, CDCl_3_) *δ* = 143.75, 128.50, 122.57, 126.51, 77.33, 77.01, 76.69. 76.25. Mass Calculated for C_13_H_12_O 184.09, Observed [M + H]^+^ = 185.

The synthesis of the similar compound exists, however, the compound is prepared from Fenofibrate by the hydrolysis of the isopropyl group followed by the reduction of both ketonic group as well as carboxylic group by BH_3_ in THF followed by the oxidation by Dess-Martin but no selectivity was reported.

In summary, we have developed a facile and highly chemoselective direct reduction of carboxylic acid to alcohol in presence of ketone. The reaction is carried out using easily accessible and cost effective reagents under extremely mild conditions. The reduction is complete within five minutes in excellent yield and purity. The facile and chemoselective reduction is attributed to the high reactivity of the carbonic anhydride at a low temperature. This study is the first report for the direct reduction of a carboxylic acid to corresponding alcohol in presence of a ketone group. The study offers a new chemoselective protocol for the orthogonal and chemoselective reduction useful for the development of new synthetic methodologies.

## Figures and Tables

**Table 1.** Optimization of reaction conditions.

Entry	Substrate 1 mM	Mole of NaBH_4_ (mM)	Time (min)	Temperature °C)	(Derivative) % yield	Diol* % yield
1	**1**	1.1	5	−15 to −10	**(3)** 60	**(5)** 0
2	**1**	2.0	5	−15 to −10	**(3)** 100	**(5)** 0
3	**1**	4.0	5	−15 to −10	**(3)** 98	**(5)** Trace*
4	**1**	5.0	5	−15 to −10	**(3)** 98	**(5)** Trace*
5	**1**	2.0	10	−15 to −10	**(3)** 100	**(5)** 0
6	**1**	2.0	15	−15 to −10	**(3)** 100	**(5)** 0
7	**1**	2.0	15	−15 to −0	**(3)** 100	**(5)** 0
8	**1**	2.0	15	−15 to −0	**(3)** 95	**(5)** ~5*
9	**6**	2.0	5	−15 to −10	**(6)** 0	**(7)** 100
10	**8**	2.0	5	−15 to −10	**(8)** 60	**(9)** 40

* by NMR.
